# Benzimidazolium 2-(2,4-dichloro­phen­oxy)acetate monohydrate

**DOI:** 10.1107/S1600536809045899

**Published:** 2009-11-11

**Authors:** Hui-Lian Liu, Qing-Zhong Wang, Fang-Fang Jian

**Affiliations:** a Microscale Science Institute, Biology Department, Weifang University, Weifang 261061, People’s Republic of China; b Microsale Science Institute, Weifang University, Weifang 261061, People’s Republic of China

## Abstract

In the crystal of the title hydrated mol­ecular salt, C_7_H_7_N_2_^+^·C_8_H_5_Cl_2_O_3_·H_2_O, the components inter­act by way of N—H⋯O and O—H⋯O hydrogen bonds, leading to chains propagating in [100].

## Related literature

For background to 2,4-dichloro­phenoxy­acetic acid, see: Lv (1998 ▶).
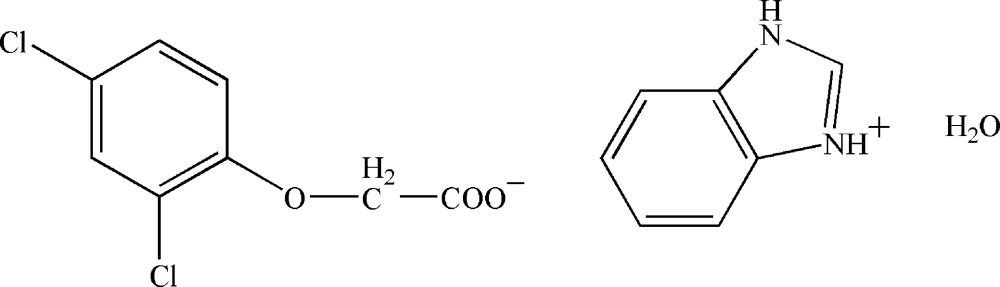


## Experimental

### 

#### Crystal data

C_7_H_7_N_2_^+^·C_8_H_5_Cl_2_O_3_^−^·H_2_O
*M* *_r_* = 357.18
Orthorhombic, 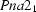

*a* = 4.9322 (10) Å
*b* = 23.808 (5) Å
*c* = 13.931 (3) Å
*V* = 1635.9 (6) Å^3^
*Z* = 4
Mo *K*α radiation
μ = 0.42 mm^−1^
*T* = 293 K
0.20 × 0.15 × 0.11 mm

#### Data collection

Bruker SMART CCD diffractometer
Absorption correction: none
13995 measured reflections
3746 independent reflections
3083 reflections with *I* > 2σ(*I*)
*R*_int_ = 0.069

#### Refinement

*R*[*F* ^2^ > 2σ(*F* ^2^)] = 0.047
*wR*(*F* ^2^) = 0.102
*S* = 0.98
3746 reflections
216 parameters
1 restraint
H atoms treated by a mixture of independent and constrained refinement
Δρ_max_ = 0.32 e Å^−3^
Δρ_min_ = −0.34 e Å^−3^
Absolute structure: Flack (1983 ▶), 1784 Friedel pairs
Flack parameter: 0.04 (5)

### 

Data collection: *SMART* (Bruker, 1997 ▶); cell refinement: *SAINT* (Bruker, 1997 ▶); data reduction: *SAINT*; program(s) used to solve structure: *SHELXS97* (Sheldrick, 2008 ▶); program(s) used to refine structure: *SHELXL97* (Sheldrick, 2008 ▶); molecular graphics: *SHELXTL* (Sheldrick, 2008 ▶); software used to prepare material for publication: *SHELXTL*.

## Supplementary Material

Crystal structure: contains datablocks global, I. DOI: 10.1107/S1600536809045899/hb5201sup1.cif

Structure factors: contains datablocks I. DOI: 10.1107/S1600536809045899/hb5201Isup2.hkl

Additional supplementary materials:  crystallographic information; 3D view; checkCIF report

## Figures and Tables

**Table 1:** Hydrogen-bond geometry (Å, °)

*D*—H⋯*A*	*D*—H	H⋯*A*	*D*⋯*A*	*D*—H⋯*A*
N1—H1*A*⋯O2^i^	0.86	1.78	2.636 (3)	179
N2—H2*A*⋯O3	0.86	1.81	2.667 (3)	172
O1*W*—H1*WA*⋯O3	0.74 (5)	2.11 (5)	2.822 (4)	160 (5)
O1*W*—H1*WB*⋯O1*W* ^ii^	0.81 (4)	1.95 (4)	2.751 (4)	173 (4)

Symmetry codes: (i) 
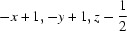
; (ii) 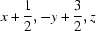.

**Fig. 1.**
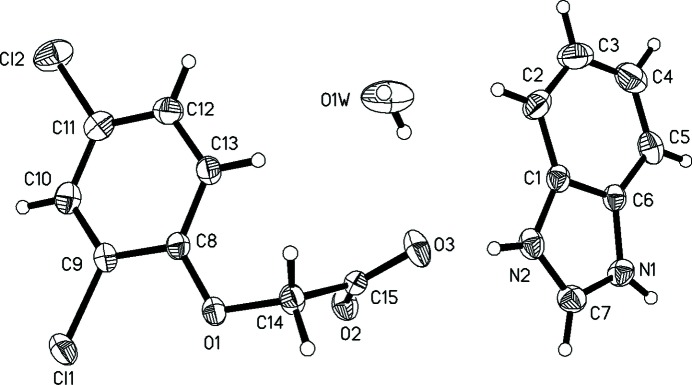
The molecular structure of (I) showing 30% probability displacement ellipsoids.

## supplementary crystallographic information

### Experimental

A mixture of 2,4-Dichlorophenoxyacetic acid 4.42 g (0.02 mol) and benzimidazole 2.4 g (0.02 mol) was stirred with ethanol (50 ml) at 367 K for 3 h. Colourless bars of (I) were obtained by recrystallization from acetone and ethanol (1:1) at room temperature.

### Refinement

H atoms were fixed geometrically and allowed to ride on their attached atoms, with C—H and N—H distances of 0.93–0.96 and 0.86 Å, and with *U*_iso_=1.2–1.5*U*_eq_.

### Crystal data

**Table:**

C_7_H_7_N_2_^+^·C_8_H_5_Cl_2_O_3_^−^·H_2_O	*F*(000) = 736
*M**_r_* = 357.18	*D*_x_ = 1.450 Mg m^−^^3^
Orthorhombic, *P**n**a*2_1_	Mo *K*α radiation, λ = 0.71073 Å
Hall symbol: P 2c -2n	Cell parameters from 2216 reflections
*a* = 4.9322 (10) Å	θ = 3.4–27.5°
*b* = 23.808 (5) Å	µ = 0.42 mm^−^^1^
*c* = 13.931 (3) Å	*T* = 293 K
*V* = 1635.9 (6) Å^3^	Bar, colorless
*Z* = 4	0.20 × 0.15 × 0.11 mm

### Data collection

**Table:**

Bruker SMART CCD diffractometer	3083 reflections with *I* > 2σ(*I*)
Radiation source: fine-focus sealed tube	*R*_int_ = 0.069
graphite	θ_max_ = 27.5°, θ_min_ = 3.4°
ω scans	*h* = −6→6
13995 measured reflections	*k* = −30→30
3746 independent reflections	*l* = −18→18

### Refinement

**Table:**

Refinement on *F*^2^	Secondary atom site location: difference Fourier map
Least-squares matrix: full	Hydrogen site location: inferred from neighbouring sites
*R*[*F*^2^ > 2σ(*F*^2^)] = 0.047	H atoms treated by a mixture of independent and constrained refinement
*wR*(*F*^2^) = 0.102	*w* = 1/[σ^2^(*F*_o_^2^) + (0.0468*P*)^2^] where *P* = (*F*_o_^2^ + 2*F*_c_^2^)/3
*S* = 0.98	(Δ/σ)_max_ < 0.001
3746 reflections	Δρ_max_ = 0.32 e Å^−^^3^
216 parameters	Δρ_min_ = −0.34 e Å^−^^3^
1 restraint	Absolute structure: Flack (1983), 1784 Friedel pairs
Primary atom site location: structure-invariant direct methods	Flack parameter: 0.04 (5)

### Special details

**Table:**

Geometry. All e.s.d.'s (except the e.s.d. in the dihedral angle between two l.s. planes) are estimated using the full covariance matrix. The cell e.s.d.'s are taken into account individually in the estimation of e.s.d.'s in distances, angles and torsion angles; correlations between e.s.d.'s in cell parameters are only used when they are defined by crystal symmetry. An approximate (isotropic) treatment of cell e.s.d.'s is used for estimating e.s.d.'s involving l.s. planes.
Refinement. Refinement of *F*^2^ against ALL reflections. The weighted *R*-factor *wR* and goodness of fit *S* are based on *F*^2^, conventional *R*-factors *R* are based on *F*, with *F* set to zero for negative *F*^2^. The threshold expression of *F*^2^ > σ(*F*^2^) is used only for calculating *R*-factors(gt) *etc*. and is not relevant to the choice of reflections for refinement. *R*-factors based on *F*^2^ are statistically about twice as large as those based on *F*, and *R*- factors based on ALL data will be even larger.

### Fractional atomic coordinates and isotropic or equivalent isotropic displacement parameters (Å^2^)

**Table:**

	*x*	*y*	*z*	*U*_iso_*/*U*_eq_	
Cl1	0.90454 (14)	0.52904 (3)	0.19850 (5)	0.05295 (18)	
Cl2	0.18254 (15)	0.69703 (3)	0.23025 (7)	0.0655 (2)	
O1	0.9973 (3)	0.57283 (7)	0.00727 (12)	0.0399 (4)	
N2	0.5521 (4)	0.57806 (9)	−0.38610 (16)	0.0433 (5)	
H2A	0.6477	0.5848	−0.3358	0.052*	
O3	0.8577 (4)	0.60800 (8)	−0.23670 (12)	0.0495 (5)	
N1	0.3939 (4)	0.53553 (8)	−0.51242 (16)	0.0424 (5)	
H1A	0.3716	0.5105	−0.5563	0.051*	
O2	0.6808 (3)	0.53999 (7)	−0.14827 (13)	0.0419 (4)	
C10	0.5471 (5)	0.61429 (10)	0.2019 (2)	0.0429 (5)	
H10A	0.5020	0.6018	0.2631	0.051*	
C15	0.8470 (4)	0.57845 (8)	−0.16206 (16)	0.0316 (4)	
C13	0.6758 (5)	0.65057 (10)	0.01831 (19)	0.0392 (5)	
H13A	0.7166	0.6629	−0.0433	0.047*	
C1	0.3528 (5)	0.61244 (10)	−0.42408 (17)	0.0378 (5)	
C8	0.8059 (4)	0.60339 (9)	0.05484 (16)	0.0345 (5)	
C14	1.0646 (5)	0.59077 (11)	−0.08771 (18)	0.0387 (5)	
H14A	1.2313	0.5724	−0.1072	0.046*	
H14B	1.0983	0.6309	−0.0867	0.046*	
C7	0.5692 (5)	0.53288 (10)	−0.4412 (2)	0.0446 (6)	
H7A	0.6888	0.5033	−0.4310	0.054*	
C11	0.4222 (5)	0.66116 (11)	0.1635 (2)	0.0437 (6)	
C12	0.4859 (5)	0.67944 (11)	0.0727 (2)	0.0450 (6)	
H12A	0.4016	0.7112	0.0477	0.054*	
C6	0.2510 (5)	0.58563 (10)	−0.50448 (18)	0.0360 (5)	
C9	0.7402 (5)	0.58614 (10)	0.14810 (17)	0.0366 (5)	
C2	0.2556 (6)	0.66476 (10)	−0.3953 (2)	0.0517 (7)	
H2B	0.3238	0.6831	−0.3415	0.062*	
C4	−0.0475 (6)	0.66061 (14)	−0.5315 (3)	0.0641 (8)	
H4A	−0.1831	0.6780	−0.5671	0.077*	
C5	0.0468 (6)	0.60888 (13)	−0.5602 (2)	0.0511 (7)	
H5A	−0.0224	0.5906	−0.6139	0.061*	
C3	0.0547 (7)	0.68769 (13)	−0.4503 (3)	0.0673 (9)	
H3A	−0.0161	0.7225	−0.4331	0.081*	
O1W	0.8508 (6)	0.72439 (12)	−0.1984 (3)	0.0798 (9)	
H1WA	0.831 (9)	0.696 (2)	−0.219 (4)	0.111 (19)*	
H1WB	0.995 (8)	0.7405 (17)	−0.194 (3)	0.088 (14)*	

### Atomic displacement parameters (Å^2^)

**Table:**

	*U*^11^	*U*^22^	*U*^33^	*U*^12^	*U*^13^	*U*^23^
Cl1	0.0616 (4)	0.0589 (4)	0.0383 (3)	0.0105 (3)	−0.0035 (3)	0.0137 (3)
Cl2	0.0578 (4)	0.0584 (4)	0.0802 (6)	0.0021 (3)	0.0287 (4)	−0.0082 (4)
O1	0.0435 (9)	0.0492 (9)	0.0271 (8)	0.0090 (7)	−0.0022 (7)	0.0020 (7)
N2	0.0522 (12)	0.0432 (11)	0.0344 (11)	−0.0106 (10)	−0.0063 (9)	0.0002 (9)
O3	0.0647 (12)	0.0488 (10)	0.0350 (9)	−0.0190 (8)	−0.0114 (8)	0.0130 (7)
N1	0.0489 (11)	0.0373 (10)	0.0410 (12)	−0.0052 (9)	−0.0017 (10)	−0.0081 (9)
O2	0.0464 (10)	0.0415 (9)	0.0378 (9)	−0.0093 (7)	−0.0024 (8)	0.0054 (7)
C10	0.0419 (12)	0.0494 (13)	0.0373 (12)	−0.0089 (10)	0.0023 (12)	−0.0011 (11)
C15	0.0352 (10)	0.0321 (10)	0.0274 (11)	0.0031 (8)	0.0026 (9)	−0.0011 (8)
C13	0.0471 (12)	0.0393 (11)	0.0313 (12)	−0.0005 (10)	−0.0020 (10)	0.0019 (10)
C1	0.0457 (12)	0.0354 (11)	0.0321 (12)	−0.0084 (10)	0.0051 (10)	0.0021 (9)
C8	0.0356 (11)	0.0380 (11)	0.0300 (11)	−0.0005 (9)	−0.0054 (10)	−0.0027 (9)
C14	0.0354 (12)	0.0497 (13)	0.0310 (11)	0.0018 (10)	−0.0023 (10)	0.0029 (10)
C7	0.0502 (14)	0.0363 (12)	0.0474 (15)	−0.0031 (10)	−0.0030 (12)	−0.0002 (11)
C11	0.0376 (12)	0.0434 (13)	0.0501 (15)	−0.0058 (10)	0.0041 (11)	−0.0061 (11)
C12	0.0462 (13)	0.0397 (12)	0.0491 (16)	0.0029 (10)	−0.0018 (12)	0.0007 (11)
C6	0.0377 (12)	0.0382 (11)	0.0322 (12)	−0.0057 (9)	0.0012 (9)	0.0008 (9)
C9	0.0391 (11)	0.0419 (12)	0.0288 (12)	−0.0036 (10)	−0.0022 (10)	0.0019 (9)
C2	0.0674 (17)	0.0394 (14)	0.0482 (16)	−0.0074 (13)	0.0174 (14)	−0.0081 (12)
C4	0.0554 (16)	0.0667 (19)	0.070 (2)	0.0127 (14)	0.0073 (16)	0.0225 (17)
C5	0.0493 (14)	0.0633 (18)	0.0409 (15)	−0.0074 (13)	−0.0075 (13)	0.0076 (13)
C3	0.078 (2)	0.0415 (15)	0.083 (3)	0.0100 (14)	0.023 (2)	0.0027 (16)
O1W	0.0654 (16)	0.0463 (13)	0.128 (3)	0.0020 (12)	0.0147 (17)	0.0039 (14)

### Geometric parameters (Å, °)

**Table:**

Cl1—C9	1.731 (2)	C1—C6	1.384 (3)
Cl2—C11	1.729 (3)	C1—C2	1.394 (3)
O1—C8	1.364 (3)	C8—C9	1.401 (3)
O1—C14	1.429 (3)	C14—H14A	0.9700
N2—C7	1.324 (3)	C14—H14B	0.9700
N2—C1	1.384 (3)	C7—H7A	0.9300
N2—H2A	0.8600	C11—C12	1.375 (4)
O3—C15	1.257 (3)	C12—H12A	0.9300
N1—C7	1.318 (3)	C6—C5	1.387 (4)
N1—C6	1.390 (3)	C2—C3	1.366 (4)
N1—H1A	0.8600	C2—H2B	0.9300
O2—C15	1.244 (3)	C4—C5	1.376 (4)
C10—C11	1.383 (4)	C4—C3	1.396 (5)
C10—C9	1.385 (3)	C4—H4A	0.9300
C10—H10A	0.9300	C5—H5A	0.9300
C15—C14	1.520 (3)	C3—H3A	0.9300
C13—C12	1.387 (3)	O1W—H1WA	0.73 (5)
C13—C8	1.390 (3)	O1W—H1WB	0.81 (4)
C13—H13A	0.9300		
			
C8—O1—C14	116.82 (18)	N1—C7—N2	110.9 (2)
C7—N2—C1	107.7 (2)	N1—C7—H7A	124.6
C7—N2—H2A	126.2	N2—C7—H7A	124.6
C1—N2—H2A	126.2	C12—C11—C10	120.7 (3)
C7—N1—C6	108.3 (2)	C12—C11—Cl2	119.7 (2)
C7—N1—H1A	125.9	C10—C11—Cl2	119.7 (2)
C6—N1—H1A	125.9	C11—C12—C13	120.0 (2)
C11—C10—C9	119.2 (3)	C11—C12—H12A	120.0
C11—C10—H10A	120.4	C13—C12—H12A	120.0
C9—C10—H10A	120.4	C1—C6—C5	122.2 (2)
O2—C15—O3	124.6 (2)	C1—C6—N1	106.0 (2)
O2—C15—C14	120.1 (2)	C5—C6—N1	131.8 (2)
O3—C15—C14	115.25 (19)	C10—C9—C8	121.3 (2)
C12—C13—C8	120.8 (2)	C10—C9—Cl1	118.83 (19)
C12—C13—H13A	119.6	C8—C9—Cl1	119.89 (18)
C8—C13—H13A	119.6	C3—C2—C1	116.4 (3)
N2—C1—C6	107.1 (2)	C3—C2—H2B	121.8
N2—C1—C2	131.5 (2)	C1—C2—H2B	121.8
C6—C1—C2	121.4 (2)	C5—C4—C3	121.8 (3)
O1—C8—C13	124.9 (2)	C5—C4—H4A	119.1
O1—C8—C9	117.0 (2)	C3—C4—H4A	119.1
C13—C8—C9	118.0 (2)	C4—C5—C6	116.1 (3)
O1—C14—C15	114.17 (19)	C4—C5—H5A	122.0
O1—C14—H14A	108.7	C6—C5—H5A	122.0
C15—C14—H14A	108.7	C2—C3—C4	122.1 (3)
O1—C14—H14B	108.7	C2—C3—H3A	118.9
C15—C14—H14B	108.7	C4—C3—H3A	118.9
H14A—C14—H14B	107.6	H1WA—O1W—H1WB	126 (4)

### Hydrogen-bond geometry (Å, °)

**Table:**

*D*—H···*A*	*D*—H	H···*A*	*D*···*A*	*D*—H···*A*
N1—H1A···O2^i^	0.86	1.78	2.636 (3)	179
N2—H2A···O3	0.86	1.81	2.667 (3)	172
O1W—H1WA···O3	0.74 (5)	2.11 (5)	2.822 (4)	160 (5)
O1W—H1WB···O1W^ii^	0.81 (4)	1.95 (4)	2.751 (4)	173 (4)

Symmetry codes: (i) −*x*+1, −*y*+1, *z*−1/2; (ii) *x*+1/2, −*y*+3/2, *z*.
